# Current Practice in Diagnosis and Treatment of Growth Hormone Deficiency in Childhood: A Survey from Turkey

**DOI:** 10.4274/jcrpe.1794

**Published:** 2015-03-05

**Authors:** Şükran Poyrazoğlu, Teoman Akçay, İlknur Arslanoğlu, Mehmet Emre Atabek, Zeynep Atay, Merih Berberoğlu, Abdullah Bereket, Aysun Bideci, İffet Bircan, Ece Böber, Şule Can, Yaşar Cesur, Şükran Darcan, Korcan Demir, Bumin Dündar, Betül Ersoy, İhsan Esen, Ayla Güven, Cengiz Kara, Mehmet Keskin, Selim Kurtoğlu, Nihal Memioğlu, Mehmet Nuri Özbek, Tolga Özgen, Erkan Sarı, Zeynep Şıklar, Enver Şimşek, Serap Turan, Ediz Yeşilkaya, Bilgin Yüksel, Feyza Darendeliler

**Affiliations:** 1 İstanbul University, İstanbul Faculty of Medicine, Department of Pediatric Endocrinology, İstanbul, Turkey; 2 Bakırköy Dr. Sadi Konuk Research and Training Hospital, Clinic of Pediatric Endocrinology, İstanbul, Turkey; 3 Düzce University Faculty of Medicine, Department of Pediatric Endocrinology, Düzce, Turkey; 4 Necmettin Erbakan University Faculty of Medicine, Department of Pediatric Endocrinology, Konya, Turkey; 5 Marmara University Faculty of Medicine, Department of Pediatric Endocrinology, İstanbul, Turkey; 6 Ankara University Faculty of Medicine, Department of Pediatric Endocrinology, Ankara, Turkey; 7 Gazi University Faculty of Medicine, Department of Pediatric Endocrinology, Ankara, Turkey; 8 Akdeniz University Faculty of Medicine, Department of Pediatric Endocrinology, Antalya, Turkey; 9 Dokuz Eylül University Faculty of Medicine, Department of Pediatric Endocrinology, İzmir, Turkey; 10 Tepecik Educational and Research Hospital, Clinic of Pediatric Endocrinology, İzmir, Turkey; 11 Bezmialem Vakıf University Faculty of Medicine, Department of Pediatric Endocrinology, İstanbul, Turkey; 12 Ege University Faculty of Medicine, Department of Pediatric Endocrinology, İzmir, Turkey; 13 Dr. Behçet Uz Children Disease and Surgery Training and Research Hospital, Clinic of Pediatric Endocrinology, İzmir, Turkey; 14 Celal Bayar University Faculty of Medicine, Department of Pediatric Endocrinology, Manisa, Turkey; 15 Fırat University Faculty of Medicine, Department of Pediatric Endocrinology, Elazığ, Turkey; 16 Göztepe Educational and Research Hospital, Clinic of Pediatric Endocrinology, İstanbul, Turke; 17 Ondokuz Mayıs University Faculty of Medicine, Department of Pediatric Endocrinology, Samsun, Turkey; 18 Gaziantep University Faculty of Medicine, Department of Pediatric Endocrinology, Gaziantep, Turkey; 19 Erciyes University Faculty of Medicine, Department of Pediatric Endocrinology, Kayseri, Turkey; 20 American Hospital, Clinic of Pediatric Endocrinology, İstanbul, Turkey; 21 Diyarbakır Children’s State Hospital and Diyarbakır Training and Research Hospital, Diyarbakır, Turkey; 22 Gülhane Military Medical Academy, Department of Pediatric Endocrinology, Ankara, Turkey; 23 Osman Gazi University Faculty of Medicine, Department of Pediatric Endocrinology, Eskişehir, Turkey; 24 Çukurova University Faculty of Medicine, Department of Pediatric Endocrinology, Adana, Turkey

**Keywords:** Survey, Growth hormone deficiency, childhood

## Abstract

**Objective:**

Approaches to diagnosis and treatment of growth hormone deficiency (GHD) in children vary among countries and even among centers in the same country. This survey, aiming to facilitate the process of preparing the new consensus on GHD by the Turkish Pediatric Endocrinology and Diabetes Society, was designed to evaluate the current practices in diagnosis and treatment of GHD in different centers in Turkey.

**Methods:**

A questionnaire covering relevant items for diagnosis and treatment of GHD was sent out to all pediatric endocrinology centers.

**Results:**

Twenty-four centers returned the questionnaire. The most frequently used GH stimulation test was L-dopa, followed by clonidine. Eighteen centers used a GH cut-off value of 10 ng/mL for the diagnosis of GHD; this value was 7 ng/mL in 4 centers and 5 ng/mL in 2 centers. The most frequently used assay was immunochemiluminescence for determination of GH, insulin-like growth factor-1 and insulin-like growth factor binding protein-3 concentrations. Sex steroid priming in both sexes was used by 19 centers. The most frequently used starting dose of recombinant human GH (rhGH) in prepubertal children was 0.025-0.030 mg/kg/day and 0.030-0.035 mg/kg/day in pubertal children. Growth velocity was used in the evaluation for growth response to rhGH therapy in all centers. Anthropometric measurements of patients every 3-6 months, fasting blood glucose, bone age and thyroid panel evaluation were used by all centers at follow-up. Main indications for cessation of therapy were decreased height velocity and advanced bone age. Fourteen centers used combined treatment (rhGH and gonadotropin-releasing analogues) to increase final height.

**Conclusion:**

Although conformity was found among centers in Turkey in current practice, it is very important to update guideline statements and to modify, if needed, the approach to GHD over time in accordance with new evidence-based clinical studies.

## INTRODUCTION

The diagnosis and treatment of growth hormone deficiency (GHD) are important and challenging issues. Several prevailing guidelines for the diagnosis and treatment of GHD in children have been published (1,2,3,4,5). However, differences in diagnostic procedures and treatment strategies between countries and even among centers in the same country continue to exist (6,7). Recently, the Turkish Pediatric Endocrinology and Diabetes Society has been preparing new consensus guidelines for diagnostic procedures and treatment in children with GHD. We believe that this survey, based on feedback obtained from the clinicians and the results of which are presented below, is timely and will be helpful in preparing the new consensus and incorporating the recent practices into the consensus statement.

We aimed to evaluate how diagnosis and treatment of childhood GHD is currently carried out in day-to-day routine among Turkish pediatric endocrinologists.

## METHODS

A questionnaire was sent out via internet to all pediatric endocrinology centers (n=44). Each center has at least one pediatric endocrinologist who has completed the fellowship and certificate programs. There were two general sections in the questionnaire, the first part relating to diagnosis and the second to treatment of GHD in childhood. The contents of the questionnaire are shown in Table 1. Each question could be answered by choosing several predefined options or if not suitable by a comment. Data were reported as percentage of respondents.

## RESULTS

Twenty-four centers (55%), (19 medical schools, 4 public hospitals and 1 private hospital) responded to the questionnaire. Thus, our survey appears to be representative of current practice within Turkey. The estimated total number of patients put on recombinant human GH (rhGH) treatment per year by the respondents was 903 [mean 43±20 (range=4-80)]. Distribution of centers by number of patients followed was: 80-60 patients/year, 20.8% (5 centers); 59-40 patients/year, 29.2% (7 centers); 39-20 patients/year, 33.3% (8 centers) and <20 patients/year, 16.7% (4 centers).

### Diagnosis of Growth Hormone Deficiency

The diagnosis of GHD was always confirmed by two GH stimulation tests by all of the respondents. Choice of GH stimulation tests varied according to centers (Table 2). L-dopa test was the most frequently used GH stimulation test. Clonidine test was used as the second frequent test. Insulin tolerance test (ITT) and glucagon test were other tests used for stimulation.

Eighteen centers (75%) used a cut-off value of GH peak of 10 ng/mL for the assessment of GHD; this value was 7 ng/mL in 4 centers (16.7%) and 5 ng/mL in two centers (8.3%).

### Diagnosis of Growth Hormone Deficiency in the Newborn Period

Results of random spontaneous GH levels in the newborn period was reported by 18 respondents (75%). Normality was defined as a GH concentration of 20 ng/mL by 10 respondents (55.6%), whereas 3 respondents (16.7%) used reference values similar to those of their GH stimulation testing, as 10 ng/mL. Another 5 respondents (27.8%) reported this value as 7 ng/mL.

### Assay for Growth Hormone

The most frequently used GH assays were immunochemiluminescent assay (ICMA) (n=21, 87.5%), immunoradiometric assay (IRMA) (n=1, 4.2%) and radiommunoassay (RIA) (n=2, 8.3%). When asked which brands of assay were used for GH analysis, all respondents were aware of the brands of the assay they were using.

### Insulin-Like Growth Factor-1 (IGF-1) Assay

When we asked what type of IGF-1 assay was used, 8.3% (n=2) did not know which assay was used. The most frequently used type of IGF-1 assays were ICMA (n=17, 70.8%), IRMA (n=2, 8.3%) and RIA (n=3, 12.5%). When brands of assay material used for their IGF-1 analysis were asked, 7 respondents (29.2%) did not answer this question and 2 respondents (8.3%) did not know what assay material they were using.

### IGF binding protein-3 (IGFBP-3) Assay

The most frequently used IGFBP-3 assays were reported to be ICMA (n=17, 70.8%), IRMA (n=3, 12.5%) and RIA (n=1, 4.2%). One center did not perform IGFBP-3 assays and the other two centers did not know what assay they were using. Nine respondents (37.5%) did not know the brand of the assay they were using.

### Sex Steroid Priming

Sex steroid priming for both sexes in pre- and peripubertal patients was used by 19 respondents (79.2%), whereas 5 of the centers did not prime with a sex steroid before GH stimulation testing. Three centers did not state the criteria which they used. Six centers used bone age to decide whether or not priming was necessary and other centers used chronological age (Table 3). For priming, ethinyl estradiol was used (n=9, 47.4%) most frequently in the girls. Others used 17β-estradiol (n=8, 42.1%) and estradiol valerate (n=2, 10.5%). For boys, intramuscular administration of long-acting testosterone esters (Sustanon® or Testoviron®) was used in all centers. The most frequently used dose was 100 mg IM, administered 3-7 days before GH stimulation testing.

### Treatment of Growth Hormone Deficiency

All respondents reported that they adjusted the starting rhGH dose according to body weight. The starting dose of rhGH for prepubertal children was 0.025-0.030 mg/kg/day in 15 centers (62.5%) and 0.031-0.035 mg/kg/day in 9 centers (37.5 %). For pubertal children, the dose most commonly used was 0.030-0.035 mg/kg/day in 16 centers (66.7%), 4 centers (16.7%) used a dose lower than 0.03 mg/kg/day and 4 centers (16.7%) used a dose higher than 0.035 mg/kg/day. rhGH is administered subcutaneously in the evening on a daily basis in all centers.

All respondents indicated that they performed a cerebral magnetic resonance (MR) scanning in GHD children at the time of diagnosis. Fundoscopic examination of patients was performed in 7 (29.2%) centers before onset of rhGH treatment.

### Evaluation of Growth Response of Patients on rhGH Therapy

All centers used growth velocity and delta height standard deviation score (∆ Ht SDS) on an annual basis in the evaluation of growth response. The response was also evaluated according to IGF-1 (n=6, 25%) and according to bone age (n=3, 12.5%). Dose adjustment was made according to growth velocity in all centers and 18 centers also used IGF-1 levels to adjust the dose of rhGH.

### Monitoring of rhGH Therapy

Selection and frequency of the most common laboratory screening tests during therapy are shown in Table 4. All centers evaluated anthropometric measurements of patients every 3-6 months of therapy. Fasting blood glucose, left hand and wrist x-ray and thyroid panel evaluation were used by all centers.

### Cessation of rhGH Therapy

GH therapy was stopped primarily according to height velocity in all centers and bone age in 22 centers (92%). Six centers did not give absolute values of height velocity cut-off to stop GH therapy. Height velocity of less than 2 cm per year was used in 12 (50%) centers, 5 cm in 4 (16.6%) centers and 4 cm in 2 (8.3%) centers. Fourteen centers, which used bone age for cessation of GH treatment, did not report cut-off value for bone age. Bone age cut-off values of greater than 16 years in boys and 14 years in girls were used in 8 (33.3%) centers. Attaining a 25th percentile value in height for age (n=12, 50%) and attaining target height range (n=6, 25%) were the other criteria for stopping therapy.

### Concomitant Therapy with rhGH During Puberty to Increase Growth Potential

The practice of using a combination of gonadotropin-releasing hormone analogues (GnRHa) or aromatase inhibitors (boys) and rhGH was asked in the questionnaire. Fourteen centers (58.3%) used combined treatment with rhGH and GnRHa to increase the final height by delaying puberty and slowing bone maturation in girls and 7 centers (29.2%) used aromatase inhibitors in boys.

### Induction of Puberty

In boys with hypogonadotropic hypogonadism, puberty was induced according to chronological age, frequently at 13-14 years (ranging from 11 to 16 years). Six centers did not use chronological age for puberty induction and bone age was used as the criterion for induction. The most commonly used bone age cut-off for puberty induction was a bone age of 12-13 years for boys.

In girls with hypogonadotropic hypogonadism, puberty was induced at 12-13 years of age (ranging from 10 to 14 years). Similar to boys, six centers did not use chronological age for puberty induction in girls and the most commonly used bone age for puberty induction was 11-12 years.

### Onset of rhGH Treatment in Idiopathic Growth Hormone Deficiency

rhGH therapy was started after obtaining a pretreatment growth velocity in idiopathic GHD in most of the centers. Most of the centers (n=17, 70.8%) started rhGH therapy after 6 months of a pretreatment follow-up period.

### Onset of rhGH Therapy in Craniopharyngioma and Other Malignancies

The period between the end of the tumor therapy and the initiation of rhGH therapy was most frequently 2 years in craniopharyngioma (n=13, 54.2%). It was 6 months in 4 centers, 1 year in 6 centers and 3 years in 1 center. The period was similar for malignancies other than craniopharyngioma. Most of the centers (n=15, 62.5%) waited for 2 years in malignancies other than craniopharyngioma. In one center, this waiting period was 6 months, it was 1 year in 3 centers and 3 years in 1 center. One center did not use rhGH treatment in malignancies and 3 centers did not have such patients.

### Side Effects of rhGH Treatment

The most common side effects during rhGH treatment reported in 12 (50%) centers were benign intracranial hypertension and slipped capital femoral epiphysis. Neoplasms were reported in 3 patients during GH treatment [osteochondroma (n=1), gastric carcinoid tumor (n=1) and malignant melanoma after optic glioma (n=1)].

## DISCUSSION

We believe that this present survey among pediatric endocrinology centers on current practice in diagnosis and treatment of GHD is of great importance in the preparation of guidelines for diagnosis and treatment of GHD in our country. We have found much conformity among centers in current practice.

Traditionally, GH stimulation tests continue to play a primary role in the diagnosis of GHD. The cut-off levels used to define GH deficiency are arbitrary. A peak stimulated GH of less than 10 ng/mL was the usual cut-off value reported by the centers. Several different stimuli (insulin, L-dopa, clonidine, glucagon, etc.) are currently being used to induce GH secretion, since they act by different mechanisms. Because the Turkish social security system, for meeting the treatment costs, requires 2 GH stimulation tests to diagnose GH deficiency, all centers have to confirm the diagnosis of GHD using two separate GH stimulation tests. Although the use of GH stimulation test varied among centers, L-dopa was the most frequently used test. The majority of the centers also used clonidine in the first testing. ITT was not among the two most commonly used tests for the diagnosis of GHD, being used in only 2 (8.3%) centers as a first test and in 5 (20.8%) centers as a second test. GH stimulation tests remain the subject of much controversy and there are significant issues concerning the validity and reproducibility of GH testing. The low specificity or sensitivity of these tests greatly reduces their diagnostic reliability (8,9). Very low reproducibility has been reported for all stimulation tests in children (10,11,12,13). Although ITT is accepted as the gold standard for the clinical practice (14), 50% of short prepubertal children with normal height velocity, were also found to have peak serum GH concentrations during ITT below 10 ng/mL (15). Tillman et al (16) reported that sensitivity of the GH stimulation test using a cut-off of 7.5 ng/mL was 73% with a specificity of 85% in a group of children diagnosed with GHD based on clinical and auxological data, along with a group of short children without GHD. Ghigo et al (8) demonstrated, in a study on 472 children without a diagnosis of GHD and with a normal growth velocity, that the GH response to a number of stimuli often failed to raise the GH levels above 7-10 ng/mL.

Twenty-three and 49.1% of the subjects given ITT, L-dopa and clonidine tests have shown a GH response lower than 7 and 10 ng/mL, respectively. A number of other studies involving GH stimulation tests in healthy adults and children with normal growth velocity have demonstrated similar rates of false positive results when the cut-off is 10 ng/mL (17,18,19).

In our survey, cut-off values for GH stimulation testing clustered around 10 ng/mL. The arbitrary cut-off level of 10 ng/mL was based on GH values measured by competitive polyclonal radioimmunoassay (20). New monoclonal fully automated non-isotopic assays are able to detect significantly lower GH levels. GH levels using newer assays are two to threefold lower than with older assays (21,22). The cut-off of 10 ng/mL was not useful when monoclonal kits were used. Cut-off for serum GH values as different as 5-10 ng/mL have been reported. The Canadian Pediatric Endocrine group has reached a consensus defining 8 ng/mL as the cut-off level for the diagnosis of GHD in children using the Immulite 2000 assay (23). Tillman et al (16) reported that a peak GH value less than 7.5 ng/mL following a GH provocative test is the most efficient standard for GHD diagnosis. It has also been suggested that the cut-off should be at 7 ng/mL (24). Similarly, serum random GH levels in a polyclonal radioimmunoassay of less than 20 ng/mL was suggested as a criterion for GHD in the newborn period. Binder et al (25) reported that a single randomly taken GH level of <7 ng/mL confirms the diagnosis of severe GHD with 100% sensitivity and 98% specificity by using a highly sensitive GH ELISA. Appropriate adjustment of cut-off levels to GH assay is a necessary requirement to avoid GHD misdiagnosis in non-GHD short patients. When considering the lower GH values measured by the current commonly used immunometric assays, an appropriately lower cut-off level for the diagnosis of GHD for both the newborn period and childhood is needed. In our recently prepared guideline, we recommended a cut-off level of 7 ng/mL for both the newborn period and childhood. The endocrinologist should be aware of the assay methodology and according to the Guidelines of GH Research Society, when assay data are reported, the method should be clearly indicated (2). However, a survey conducted on European Society for Paediatric Endocrinology (ESPE) members in 2002 revealed that only 63% of endocrinologist knew what GH assay they were using (6). Similarly, in the US survey among pediatric endocrinologists in 1995, it was found that only 80% had knowledge on the type of GH assay they used (7). It was good to find that all the respondents in our survey knew which GH assay they were using and most of the respondents also knew which IGF-1 and IGFBP-3 assays they were using.

There is no consensus regarding sex steroid priming for GH stimulation tests. Although some studies showed that normal prepubertal children may falsely fail GH stimulation tests (26,27), other studies reported that pubertal stage did not affect GH response to stimulation (8,28). Sex steroid priming in boys and girls was used by 79.2% of the respondents and as expected, there were discrepancies with respect to sex steroid priming criteria among centers.

In our survey, the most frequently reported starting doses of rhGH (0.025 to 0.035 mg/kg/day) were compatible with the recommended doses in international consensus statements and similar to the doses reported by ESPE members (0.025-0.033 mg/kg/day), but were lower than the doses reported by 68% of American pediatric endocrinologists (0.043 mg/kg/day) (1,2,3,4,6,7).

rhGH dose adjustment was primarily based on growth velocity as recommended by other consensus publications and all centers in our survey evaluated response and change in height velocity every 3-6 months. It is recommended that rhGH should be stopped when growth velocity falls to less than 2 cm/yr and bone age is greater than 16 years in boys or 14 years in girls (2). Cessation of rhGH therapy was primarily done according to height velocity and bone age in all our centers. Growth velocity accepted by our social security system to continue rhGH therapy is 5 cm/yr and the accepted cut-off for bone age is similar to other consensus results. The Turkish social security system allows using GH until attainment of a height of 25th percentile for age according to national growth references.

In our survey, fasting blood glucose, left hand and wrist x-ray and thyroid panel were always used by all centers. Lipid profiles, hemoglobin A1c and serum cortisol level were not routinely measured in most of the centers during rhGH therapy. It is recommended that glucose metabolism be assessed in all patients before and during rhGH replacement (2,29). Most of the surveillance data do not indicate an increased incidence of type 1 diabetes associated with rhGH treatment, but an increased risk of type 2 diabetes, especially in the subgroups of patients at risk of diabetes such as Turner’s syndrome, Prader-Willi syndrome and intrauterine growth retardation. rhGH increases the extrathyroidal conversion of thyroxine to triiodothyronine and may as such unmask incipient hypothyroidism. rhGH may also reduce the bioavailability of cortisol through an enhanced conversion of cortisol to cortisone. The possibility of adrenocorticotropic hormone insufficiency to be unmasked during rhGH replacement should be considered. Regular screening for lipoprotein, liver enzymes and complete blood count in patients on rhGH therapy is not recommended. Fundoscopic examination should be performed before the initiation of rhGH treatment for benign intracranial hypertension and repeated when clinically indicated (29). According to our survey, fundoscopic examination was not performed in all centers.

Adequate management of puberty in children with GHD and hypogonadotropic hypogonadism is very important. Puberty should be induced at an appropriate time to ensure normal pubertal development without compromising final height in patients with multiple pituitary hormone deficiencies (MPHD). In our survey, most of the centers preferred to induce puberty at the upper limit of normal age of onset of puberty. These results are very comparable to the results of the ESPE survey (6,30). According to the Growth Hormone Research Society Consensus (2), in MPHD patients in whom puberty does not occur spontaneously, puberty should be initiated at the appropriate time after discussion with the patient. Although not routinely recommended, to improve final height prognosis in children with GHD who are entering into a normally timed puberty and have a poor predicted adult height, combined treatment with GnRHa and rhGH may be given in selected cases to arrest pubertal development, to slow bone maturation and to prolong the duration of GH treatment. In our survey, combined therapy with rhGH to delay puberty was preferred in 14 centers in girl patients with normally timed puberty.

In summary, although we have presented mostly similarities in the current practice in the diagnosis and treatment of childhood GHD among Turkish pediatric endocrinologists compared to international consensus statements, there are some clinical applications that have to be improved in current practice. The items that require more attention are the cut-off values for the diagnosis of GHD, the management of GHD in malignant tumors and establishment of criteria for discontinuation of GH therapy at follow-up. It is very important to revise guideline statements and to modify, if needed, the approach to GHD over time.

## Figures and Tables

**Table 1:**
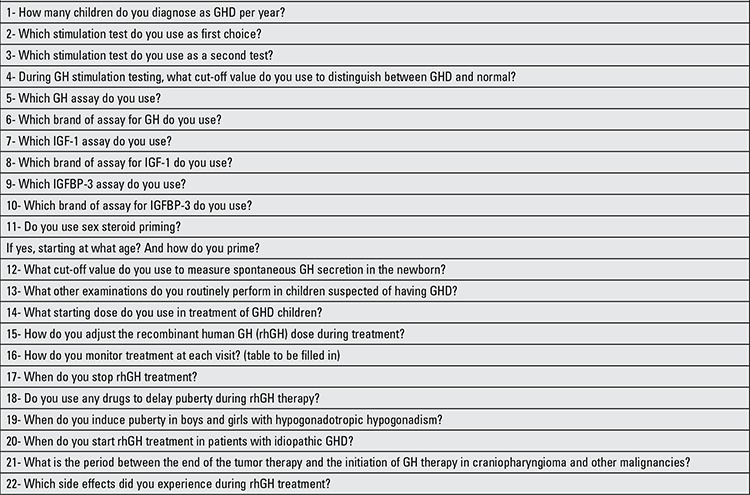
Questions asked in the questionnaire on diagnosis and treatment of childhood growth hormone deficiency (GHD) among Turkish pediatric endocrinologist

**Table 2:**
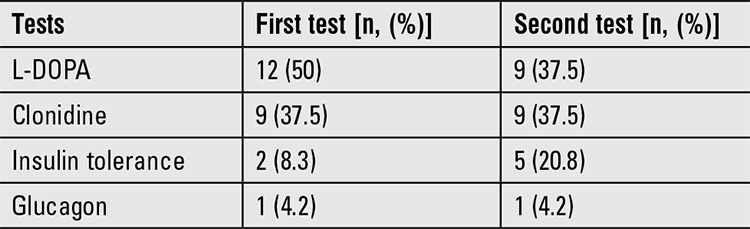
Frequency of growth hormone stimulation tests used by centers

**Table 3:**
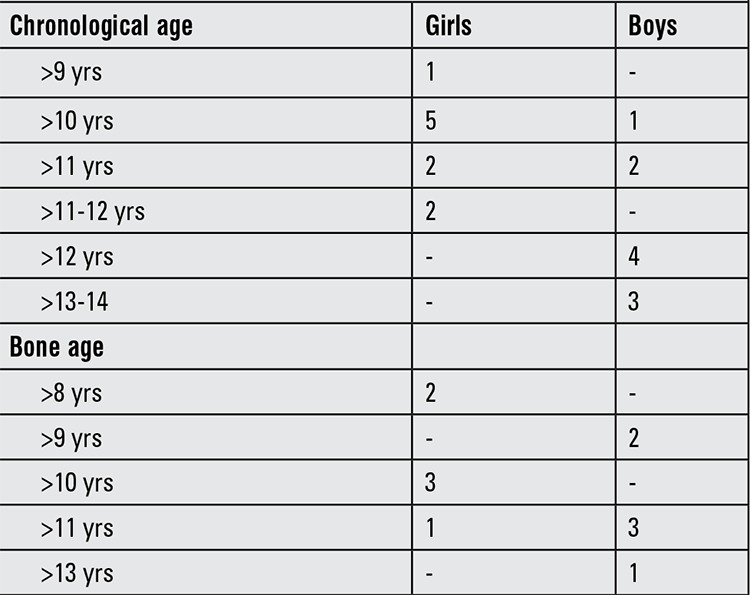
Number of centers using chronological age or bone age for sex steroid priming

**Table 4:**
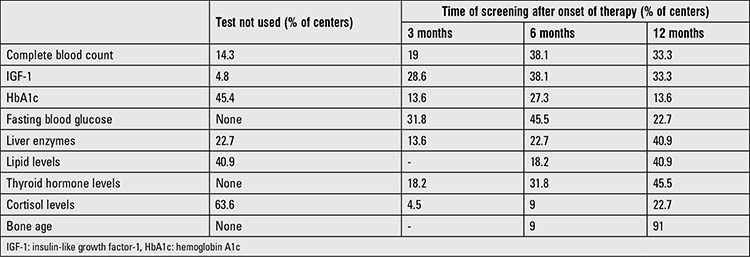
Frequency (%) of laboratory screening tests used in centers for monitoring recombinant human growth hormone therapy
